# Sleep Before and After Retirement

**DOI:** 10.1007/s40675-018-0132-5

**Published:** 2018-10-24

**Authors:** Saana Myllyntausta, Sari Stenholm

**Affiliations:** Department of Public Health, University of Turku and Turku University Hospital, Turku, Finland

**Keywords:** Retirement, Statutory retirement, Disability retirement, Sleep duration, Sleep quality, Insomnia, Aging

## Abstract

**Purpose of Review:**

In this review, we focus on the association of sleep and retirement from two perspectives. Firstly, we examine the role of sleep during the working years on retirement timing. Secondly, we examine how sleep changes during the transition to retirement.

**Recent Findings:**

Persons with sleep difficulties are more likely to retire due to health problems, such as depression and musculoskeletal disorders. Retirement, on the other hand, is associated with both increased sleep duration and decreased sleep difficulties, mainly premature awakenings and nonrestorative sleep.

**Summary:**

Promotion of sleep quantity and quality could be a potential way to support employees’ work ability and possibly even to postpone retirement, at least in relation to early retirement. Possible proposed mechanisms for the improved sleep after retirement include removal of work stress and increased flexibility in time use, which could be targeted in attempt to promote adequate and good quality of sleep already during the working years.

## Introduction

The present review focuses on the associations between sleep and retirement, a particularly timely topic, as the large baby-boomer generation is currently moving into retirement. Sleep not only is a key characteristic of health and well-being but also highly relevant in relation to work ability, as poor sleep is associated with lower work productivity, work absenteeism, and accidents [1, 2] as well as sickness absence [3–5]. Thus, adequate amount and quality of sleep are potential factors to be considered when aiming to prolong labor force participation and to postpone retirement. In regard to the association between sleep and retirement, the other interesting viewpoint is how sleep changes when people retire from work. Retirement provides an optimal window for positive changes in sleep as working hours no longer dominate sleep timings and work-related stressors are removed.

In this review, we present and summarize the current literature on the associations of sleep and retirement while considering both approaches: the role of sleep on retirement timing and the impact of retirement on sleep. We begin by exploring how the quality and duration of sleep during the working years associate with retirement timing, especially early retirement and disability retirement. Next, we will review the literature on the changes observed in both sleep difficulties and sleep duration following the transition from work to retirement. Finally, we conclude by discussing the wider societal implications of these findings.

## Sleep and Retirement Timing

Most of the literature on the association of sleep and the timing of retirement focuses on disability retirement (Table 1), a permanent deterioration of work ability due to illness, injury, or impairment, which prevents from continuing working. Those with frequent sleep problems have been found to have the highest risk for not returning to work from any disability period [6] and for subsequent disability retirement [7–11], but even occasional sleep problems have been found to predict disability retirement [10]. However, in terms of the association between sleep duration and disability retirement, the findings are somewhat contradictory. Some studies report that those workers with either short (≤ 5 h) or long (≥ 9 h) sleep are more likely to retire due to disability than those with average sleep (7–8 h) [11], while some studies have found this association only among long sleepers [9, 12] and some not at all [13]. The simultaneous examination of sleep duration and insomnia symptoms has revealed that even those with average sleep duration had a higher risk for subsequent disability retirement if they simultaneously experienced insomnia symptoms and, on the other hand, there was a much lower risk for disability retirement among short and long sleepers if frequent insomnia symptoms were not present [11]. Thus, the quality of sleep seems to play a stronger role than sleep duration in predicting the risk of work disability and disability retirement.

**Table 1:** Association of sleep duration and quality on retirement timing

First author, publication year	Study population *N*, cohort (age at baseline, % female, country)	Year, baseline	Follow-up time	Sleep measures	Retirement outcome	Main findings
Sivertsen et al. 2006 [7]	*N* = 37,308, Nord-Trøndelag Health Study [HUNT-2] (20–66 years, 53% female, Norway)	1995–1997	18–48 months	Self-reported (sleep onset or maintenance) insomnia and impaired work performance caused by sleep problems	Disability retirement	• 2.5% granted a disability pension • Insomnia with daytime work impairment was an independent risk factor for subsequent award of a disability pension
Øverland et al. 2008 [8]	*N* = 37,302, Nord-Trøndelag Health Study [HUNT-2] (20–66 years, 53% female, Norway)	1995–1997	18–48 months	Self-reported (sleep onset or maintenance) insomnia	Disability retirement	• 2.4% granted a disability pension • When compared to depression, insomnia had an equally important and independent role in predicting subsequent disability pension
Sivertsen et al. 2009 [9]	*N* = 6599, Hordaland Health Study [HUSK] (40–45 years, Norway)	1997–1999	12–48 months	Karolinska Sleep Questionnaire	Disability retirement	• 1.8% granted a disability pension • Insomnia symptoms and long sleep duration (> 8.5 h) were risk factors for disability retirement
Lallukka et al. 2011 [10]	*N* = 5986, Helsinki Health Study (40–60 years, 80% female, Finland)	2000–2002	6–8 years	Jenkins Sleep Problem Scale	Disability retirement	• 8% retired due to disability • Sleep problems predicted retirement due to all causes, mental disorders, and musculoskeletal disorders
Haaramo et al. 2012 [11]	*N* = 6042, Helsinki Health Study (40–60 years, 80% female, Finland)	2000–2002	Mean 8 years	Self-reported sleep duration during weekdays and Jenkins Sleep Problem Scale + joint variable of these	Disability retirement	• 9% retired due to disability • If insomnia symptoms were present, even those with average sleep duration had higher risk for disability retirement, while the highest risk was among those with both insomnia symptoms and short or long sleep duration
Ropponen et al. 2013 [13]	*N* = 18,979 (twin individuals), The Finnish Twin Cohort study (mean 45 years [DP due to LBD] and 38 years [no DP], 53% female, Finland)	1975 (+1981)	Mean 18 years (SD = 7)	Self-reported sleep quality and sleep length	Disability retirement (due to low back disorders)	• 2.5% granted disability pension due to low back disorder • Sleep quality and changes in sleep quality predicted disability pensions due to low back disorders
Lallukka et al. 2014 [5]	Two cohorts: *N* = 6892, Hordaland Health Study [HUSK] (40–45 years, 59% female, Norway) and *N* = 6060, Helsinki Health Study [HHS] (40–60 years, 78% female, Finland)	1997–1999 (HUSK) and 2000–2002 (HHS)	5 years (mean follow-up 2.8 years [SD = 0.9] in HUSK and 2.5 [SD = 1.4] in HHS)	Karolinska Sleep Questionnaire (in HUSK) and Jenkins Sleep Problem Scale (in HHS)	Disability retirement	• 3% retired due to disability in HUSK and 5% in HHS • Synergistic association of pain and insomnia observed with subsequent disability retirement
Canivet et al. 2014 [12]	*N* = 4319, Malmö Shoulder and Neck cohort (45–64 years, 48% female, Sweden)	1992–1994	12 years	Self-reported sleep duration during weekdays and insomnia symptoms	Disability retirement	• 12% retired due to disability • Moderate or considerable insomnia symptoms were an independent risk factor for subsequent award of a disability pension • Difficulties falling asleep associated with disability retirement due to mental disorder in men and due to cardiovascular diseases in women
Paunio et al. 2015 [14•]	*N* = 10,959, The Finnish Twin Cohort study (18–45 years, 55% female, Finland)	1975 and 1981	29 years	Self-reported sleep quality	Disability retirement	• Long-term poor sleep increased risk for depression and disability retirement due to depression
Hale et al. 2017 [15•]	*N* = 1635, The Retirement and Sleep Trajectories study (50 years [±4 years], 52% female, USA)	1988	Mean 11 years (SD = 4.7)	Insomnia symptoms	Self-reported retirement	• Early morning awakenings were associated with increased rate of earlier retirement • All insomnia symptoms predicted earlier retirement due to poor health or disability
Dong et al. 2017 [16•]	*N* = 5746, The Health and Retirement Study (50–70, 48% female, USA)	2004	2 years	Number of insomnia symptoms	Self-reported job exit	• Elevated number of insomnia symptoms was associated with job exit due to poor health

Some research has also focused on cause-specific disability retirement including retirement due to musculoskeletal disorders [10, 11, 13] and mental disorders [10–12, 14•]. Self-reported short sleep duration (≤ 5 h) and frequent insomnia symptoms have been found to predict disability retirement due to both musculoskeletal disorders and mental disorders [10, 11]. In addition, men with sleep disturbances have been observed to have a higher risk for not returning to work following a disability caused by a mental disorder or disease of musculoskeletal system than men without sleep disturbances [6]. Fairly poor or poor quality of sleep has also been found to predict subsequent disability retirement due to low back disorder [13]. In addition, those reporting insomnia symptoms and pain simultaneously were observed to have the highest risk for disability retirement, suggesting a synergistic effect of insomnia symptoms and pain [5]. Furthermore, an onset of poor sleep has been found to predict incident depression and to increase risk of disability retirement due to depression almost threefold [14•]. Based on these findings, it can be postulated that an early detection and treatment of insomnia and other sleep problems is important and could potentially help to maintain workers’ work ability and to prevent disability retirement. This would seem to be particularly important among workers retiring due to low back disorder and depression.

All of the abovementioned studies on disability retirement have obtained the retirement data from pension registers, which is regarded as highly accurate and objective data source, and usually includes information on both the exact timing and the diagnosis the disability pension is based on. There are, however, also few studies that have used self-reported job exit/retirement as an outcome instead and focused on different reasons for retirement [15•, 16•]. The findings from these two studies are consistent with those examining disability retirement as the outcome showing an association with insomnia symptoms and subsequent job exit due to poor health, but not due to other reasons. Thus, these studies strengthen the findings of poor sleep quality being related to retirement due to poor health in particular.

Despite relatively consistent findings, there are also challenges in examining the role of sleep on disability retirement. As disability retirement is always preceded by a diagnosis of a disease or chronic condition, it may be difficult to isolate the impact of sleep from other factors and health in general. It can be presumed that poor sleep may lead to deteriorated health and work ability, but other factors, such as work-related factors and health behaviors, might also mediate the association between sleep and subsequent disability retirement, or on the contrary, be the cause of the poor sleep [12]. Many of the previous studies have controlled for possible confounders, such as work stress and obesity, physical activity, and other health behavioral factors, and found the associations between poor sleep and disability retirement to attenuate, but nevertheless remain significant after these adjustments.

In addition to current literature on the associations of poor sleep quality and inadequate duration with subsequent negative outcomes in employment, such as sickness absences and early retirement, it would be worth examining whether good sleep has positive effects on employment and extending working careers beyond the statutory retirement ages. To the best of our knowledge, there is currently no knowledge on whether those who sleep well during the working years are more likely to continue working longer and, thus, to postpone their retirement. Such finding would further emphasize the importance of promoting the sleep of employees when aiming to support work ability of older employees and to prolong their labor force participation.

## Changes of Sleep During the Transition to Retirement

With increasing number of people retiring, the changes in sleep following retirement have gained more interest during the previous years and both the changes in sleep duration and sleep quality have been examined. The first studies examined retirement and sleep by comparing those already retired to those still working in cross-sectional settings [17, 18, 19••]. The main limitation with these studies is that the differences observed in sleep between workers and retirees may not be due to retirement per se, but due to differences between the individuals who retire and those who continue working. In recent years, a few longitudinal studies have emerged in which sleep of the participants has been repeatedly measured before and after retirement allowing examination of true changes during retirement transition [20, 21, 22•, 23•, 24•].

Retirement is a major life transition for the individual as daily schedules and activities are altered with working hours removed from the schedule. These changes may potentially lead to decreases in daytime structure as well as to less regular sleeping patterns. For example, it is possible to stay in bed longer in the mornings as well as to take daytime naps more freely after retirement. These potential changes in sleeping patterns could in turn lead to reduced homeostatic sleep pressure [25] and, consequently, to a poorer quality of sleep during the nights.

However, findings from recent studies suggest that retirement may have a beneficial effect on sleep quality. Longitudinal studies from French and Finland have observed, for instance, that sleep difficulties decrease substantially following retirement [20, 24•]. When changes in different types of sleep difficulties have been examined, decreases have been observed especially in premature awakenings [21, 24•] and nonrestorative sleep, that is feeling tired and worn out after the usual amount of sleep [24•]. These improvements in sleep quality have been suggested to be driven by the removal of work stress, as the observed decreases in sleep disturbances have been most prevalent among those with job strain and psychological distress before retirement [20, 24•]. Psychosocial stress is associated with poor sleep quality [26, 27•], and after retirement, the work-related stressors are removed. The improvements in sleep quality might also be due to positive changes in lifestyle and health which have been observed following retirement [28, 29] or due to the demands on health being lower after the obligation to be able to work is removed. It is also possible that the changes in sleep quality are in fact changes in how sleep is perceived and reported; for example, difficulties falling asleep and waking up during the night might not be experienced as disturbing as during the working years, as poor sleep can be compensated for by sleeping longer in the morning or by taking naps during the following day. In addition, the pressure to acquire an adequate amount of good quality sleep to be able to work on the following day is removed.

Also, changes in sleep duration during retirement transition have been examined in two recent longitudinal studies [22•, 23•]. The results show that sleep duration increases following a transition to retirement among both men and women. Highly similar levels of increases in sleep duration were observed in these studies, with the first one [22•] reporting 15, 16, and 22 min longer sleep durations 1, 2, and 3 years post-retirement among retirees than among those who continued working full time over the same period and the other one [23•] observing an increase of 22 min during a four-year period around the transition to retirement. The most pronounced increases in sleep duration following retirement have been observed to occur among those with short sleep duration (≤ 6.5 h/24 h), sleep difficulties, heavy alcohol use, and psychological distress before retirement [23•]. In addition, a study examining changes in time use following retirement observed an increase in the time used for sleeping of approximately 30 min already 3 months post-retirement [30]. One explanation for increased sleep duration may be that leisure time increases after retirement as working hours are removed, and this may allow more time being devoted to sleeping. In addition, retirement removes the pressure to sleep at fixed times, working hours no longer dominate sleeping patterns, and there is no need to use an alarm clock; thus, there is a possibility to sleep longer during the mornings. Along with increased sleep duration, later bed times and wake times have been observed [22•].

These findings of improved duration and quality of sleep after retirement are important, as by understanding how retirement affects sleep and who benefit most of retirement, it may be possible to promote positive changes in sleep before and after retirement as well. Improvements in sleep may also translate into better daily functioning [31, 32•] and quality of life [33, 34]. In fact, retirement has been associated with both decreases in mental and physical fatigue [35] and improved self-rated health [27]. A major limitation of the previous literature is its reliance on self-reported information on sleep. The only exception to the previous literature is provided by a unique case study [36•] in which the author wore an accelerometer continuously for more than three decades and observed that the difference in sleep duration between weekday and weekends/days off diminished after retirement. Repeatedly conducted accelerometer measurements would indeed provide more accurate and reliable assessment of changes in sleep duration and quality around the retirement transition. With accelerometers, it would also be possible to examine the interrelationship between sleep duration and quality. Moreover, conducting measurements on both weekday and weekend nights would allow detailed examination of changes in the timing and variability of sleep. As there are already some implications on changes to later bed times and wake times after retirement [22•], it would also be valuable to examine whether different changes are observed among people with different circadian preferences (i.e., the morning and the evening types). For example, it could be examined whether those with evening preference benefit more from retirement in relation to sleep, as after retirement, they may adjust their sleeping patterns according to their preferences which may not have been possible during the working years due to externally imposed temporal patterns.

## Conclusions

As the baby boomer generation is moving into retirement, and the old-age dependency ratio is increasing, many Western governments are aiming at prolonging the labor force participation and raising the retirement ages. Based on the reviewed literature, promoting adequate sleep quantity and quality of employees could be a potential way to support their work ability and possibly even to postpone retirement, at least in the case of early retirement and disability retirement. However, as it is not yet known whether good quality sleep has protective effects on employment, such as being able to continue in the work force beyond the statutory retirement age, further research is needed on this topic. Moreover, transitioning to retirement seems to have positive effects on sleep, and improvements have been observed in both sleep duration and quality. The removal of work stress and the increased flexibility in time use have been proposed as potential mechanisms for the improvements in sleep after retirement. Thus, better sleep among older workers could possibly be promoted by, e.g., reducing work stress and with better work-time control. To date, the research on the associations of retirement and sleep, in relation to both the timing of retirement and the changes in sleep following it, has been almost entirely conducted in Northern Europe (Finland, Sweden, and Norway) and the USA using self-reported methods. Thus, further research is needed to examine whether similar findings are observed in other cultures, countries, and working sectors with different retirement schemes and by using objective measurements of sleep.
